# Low Oxygen Saturation During Risperidone Therapy: A Multispecialty Approach

**DOI:** 10.7759/cureus.95464

**Published:** 2025-10-26

**Authors:** Evaristus C Ezema, Gideon O Idoko, Samuel Alao, Nnenna B Emejuru, Ogochukwu Agazie, Omotola Emmanuel, Cheikh S Galledou, Oluseye O Asaolu, Sanmi M Obe, Chidalu Ibeneme, Amarachi N Abanobi, Olaniyi Ogundeji, Chinenye L Aleke, Olasumbo E Fagbenle

**Affiliations:** 1 Psychiatry, Interfaith Medical Center, Brooklyn, USA; 2 Public Health, Fanshawe College, Ontario, CAN; 3 Psychiatry, Dr. Peyman Younesi (PY) Medical Group, Queens, USA; 4 Psychiatry and Behavioral Sciences, College of Medicine, Imo State University, Orlu, NGA; 5 General Practice, College of Medicine, University of Lagos, Lagos, NGA; 6 Health Information Management, Emory Johns Creek Hospital, Georgia, USA; 7 Hospital-Based Medicine, St. Christopher Iba Mar Diop College of Medicine, Dakar, SEN; 8 Critical Care, Life Flora Hospital, Johannesburg, ZAF; 9 Hospital-Based Medicine, Olabisi Onabanjo University Teaching Hospital, Sagamu, NGA; 10 Public Health, University of Toledo, Toledo, USA; 11 Health Sciences, Northern Arizona University, Flagstaff, USA; 12 Psychiatry and Behavioral Sciences, Holly Hill Hospital, Raleigh, USA; 13 Physiotherapy, University of Nigeria Nsukka, Enugu, NGA; 14 Hospital-Based Medicine, Ascension Seton Cedar Park Hospital, Cedar Park, USA

**Keywords:** low, oxygen, risperidone, saturation, therapy

## Abstract

Risperidone is an atypical antipsychotic medication commonly used in the treatment of schizophrenia and bipolar disorder in adults. Risperidone therapy is not typically associated with low oxygen saturation. However, recurrent severe acute respiratory distress and acute eosinophilic pneumonia have been documented during risperidone treatment, accompanied by low oxygen saturation and requiring respiratory support. There is a paucity of literature describing low oxygen saturation without acute respiratory compromise during risperidone therapy. Therefore, we present a rare case of low oxygen saturation in a patient with glucose-6-phosphate dehydrogenase (G6PD) deficiency and variant hemoglobin (elevated HbA₂) on risperidone therapy, who exhibited no respiratory compromise.

## Introduction

Risperidone is an atypical antipsychotic medication, commonly sold under the brand name Risperdal. It is chemically known as 3-{2-[4-(6-fluoro-1,2-benzoxazol-3-yl)piperidin-1-yl]ethyl}-2-methyl-4H,6H,7H,8H,9H-pyrido[1,2-a]pyrimidin-4-one [1]. It is primarily used for the treatment of schizophrenia and bipolar disorder in adults. It is also approved for the treatment of aggression and self-injurious behaviors associated with autistic disorder in children and adolescents aged 5 to 16 years [2]. Occasionally, risperidone is used off-label as an adjunct to antidepressants for the treatment of treatment-resistant depression [3]. In Canada, risperidone is indicated for the symptomatic treatment of aggression or psychosis in severe dementia of the Alzheimer’s type that is unresponsive to conservative, nonpharmacological management [4].

Risperidone can be administered orally or through injection. The injectable forms are long-acting and can last for two to four weeks. The mechanism of action of risperidone involves inhibition of D2 dopaminergic receptors and 5-HT2A serotonergic receptors in the brain [5]. By doing so, it reduces dopaminergic neurotransmission, thereby decreasing the positive symptoms of schizophrenia, such as delusions and hallucinations [6]. The half-life of risperidone is about 3 hours in extensive metabolizers and up to 20 hours in poor metabolizers [7]. Risperidone can cause a range of adverse effects, including weight gain, extrapyramidal symptoms, drowsiness, and occasional orthostatic hypotension [8].

Oxygen saturation, often expressed as SpO₂, reflects the amount of oxygen carried by hemoglobin in RBCs. Risperidone can have various effects on the respiratory system, potentially impacting oxygen saturation [9]. The normal level of oxygen saturation in the body ranges between 95% and 100%. Opioids and anesthetic agents are known to reduce oxygen saturation in the body. In 2017, it was reported that risperidone induced the recurrence of severe acute respiratory distress in a patient with psychotic disorders [9]. This was associated with low oxygen saturation that necessitated respiratory support.

The sedative effects of risperidone may indirectly impair breathing and reduce oxygen saturation [9]. Acute eosinophilic pneumonia has also been reported during risperidone therapy, with associated low oxygen saturation requiring respiratory support [10]. Despite these reports, the association between low oxygen saturation and risperidone therapy remains uncommon. The reported cases received acute respiratory intervention as part of the overall management plan. Typically, low oxygen saturation presents with respiratory distress and restlessness; in severe cases, confusion, dizziness, and cyanosis may occur. Therefore, we present a case of low oxygen saturation during risperidone therapy in a patient with glucose-6-phosphate dehydrogenase (G6PD) deficiency and a hemoglobin variant that did not warrant acute intervention. This study seeks to contribute to the understanding of rare cases of low oxygen saturation during risperidone therapy and to provide clinical insights that may inform both management strategies and future research.

## Case presentation

A 29-year-old Hispanic female, single, with two children, residing in an apartment with her family, unemployed, and supported by government assistance, with no significant past medical history and no history of prior trauma, and with a past psychiatric diagnosis of schizoaffective disorder, bipolar type, presented to the ED after being brought in by EMS. The activation was initiated by family members due to concerns regarding irritability, talking to herself, and hearing voices for the past 24 hours.

On evaluation, the patient could not sustain a meaningful conversation. She reported a history of auditory hallucinations for the past 28 hours, stating that she hears voices from multiple individuals telling her unpleasant things. The patient endorsed daily substance use, including alcohol, cigarettes, and marijuana, which she could not quantify. She further endorsed occasional smoking of cocaine. On physical examination, the patient's blood pressure was stable, and the heart rate was notable at 121 beats per minute. The temperature and respiratory rate were both normal. Abdominal examination was remarkable for a palpable, non-tender spleen. Oxygen saturation was 91% on room air, and oxygen supplementation was provided at 2 L/min via nasal cannula, with saturation improving to 94-96% until the patient became stable. Laboratory investigations revealed a CBC with differentials within the reference range. The blood alcohol level was 100. The complete metabolic panel showed deranged electrolytes with potassium of 3.4 mmol/L and an anion gap of 14 mmol/L. A urine toxicology screen was positive for cannabinoids and cocaine, with no other illicit substances detected.

**Figure 1:**
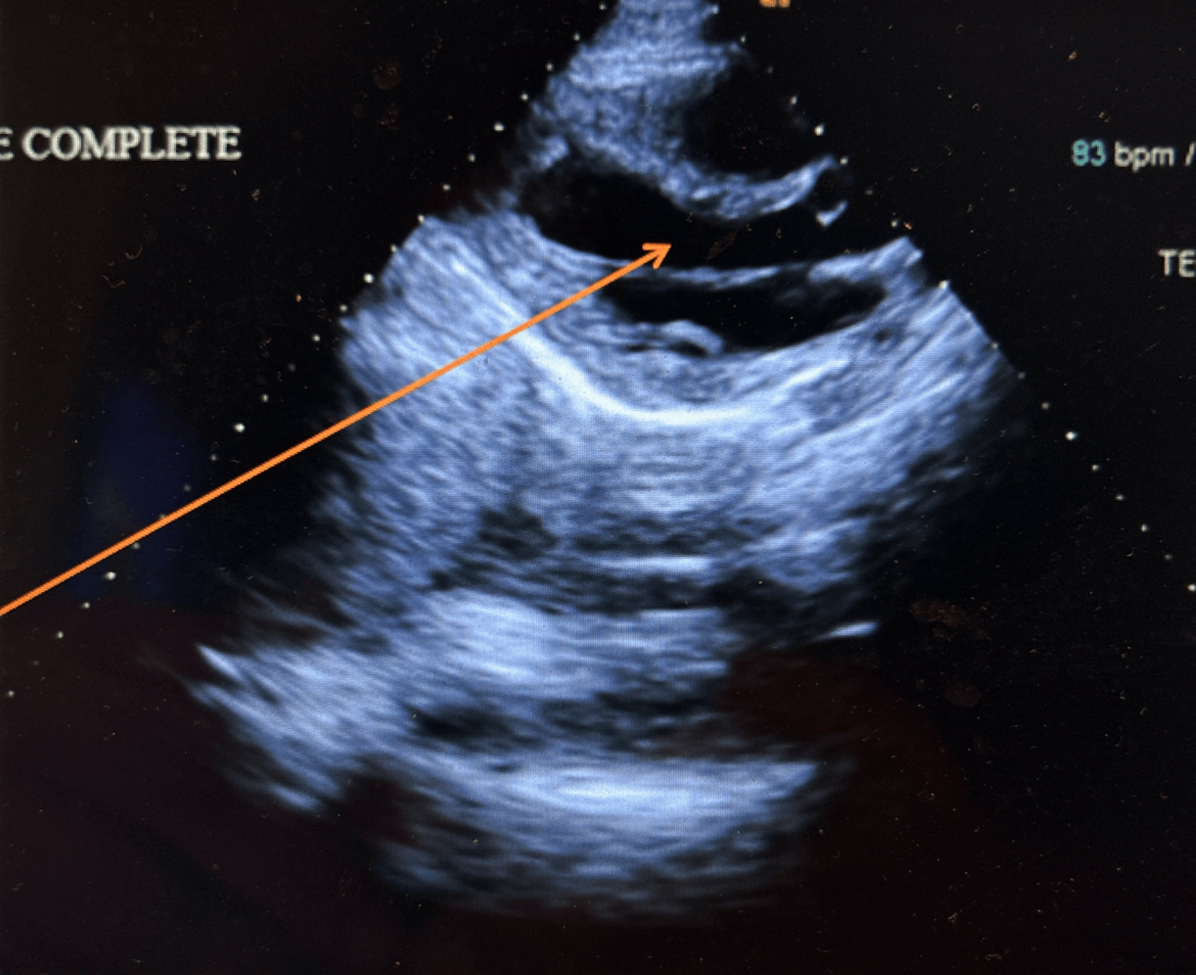
Echocardiogram showing no evidence of pericardial effusion.

**Figure 2:**
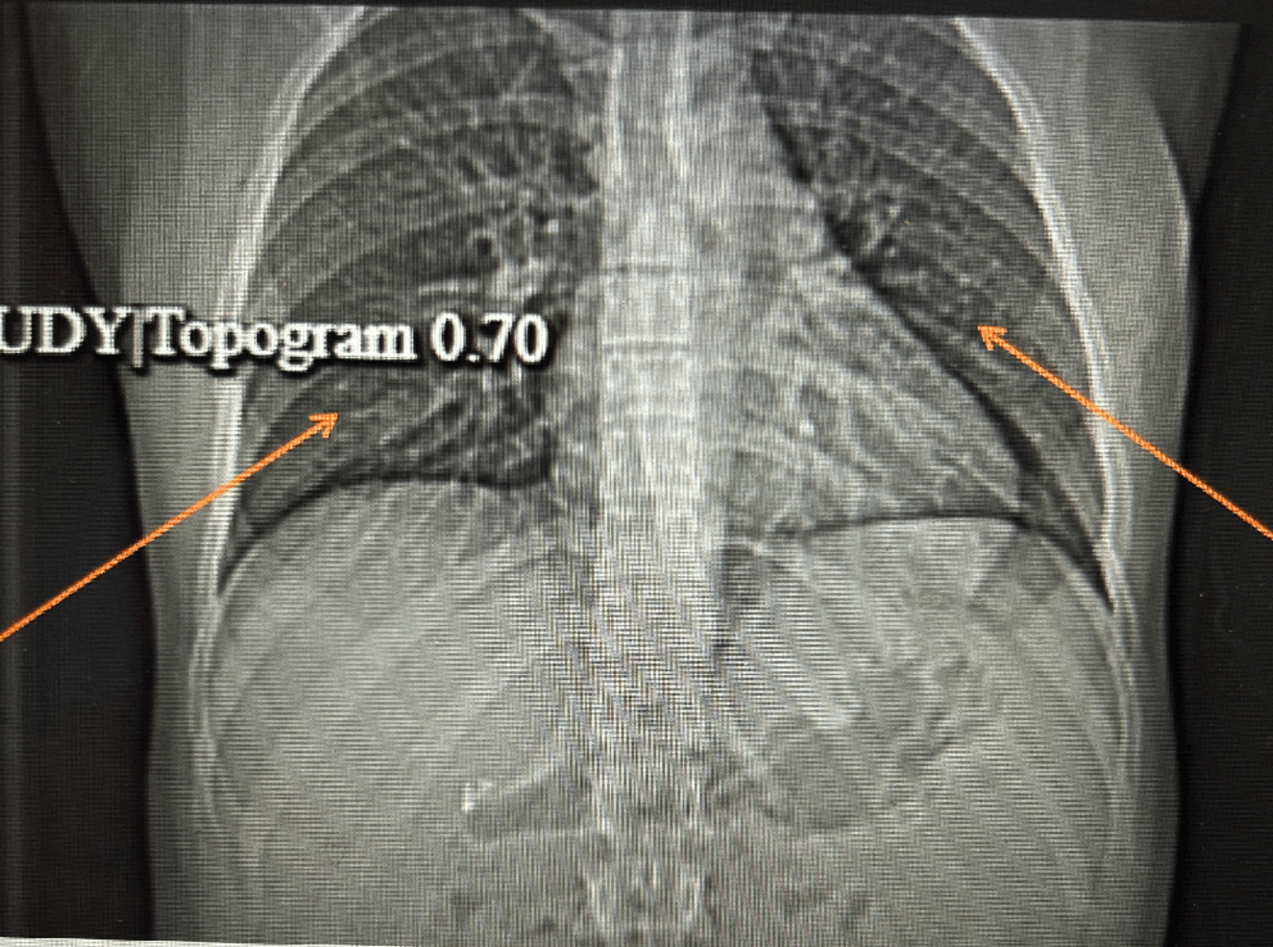
CT scan of the chest showing no evidence of pulmonary embolism.

The patient was subsequently transferred to the medical floor for management of the electrolyte derangement, where the electrolytes were corrected according to facility protocols. The patient was continued only on her home psychotropic medication, Risperidone 2 mg PO bid, and nicotine replacement therapy, which included a nicotine patch (14 mg/24 hours) and nicotine gum (2 mg PO every two hours as needed). The patient had been on Risperidone 2 mg PO bid, prescribed by a psychiatrist, for the past year. In the following days, the patient showed clinical improvement, gained more insight, and reported a significant decrease in auditory hallucinations. An attempt to wean the patient off oxygen supplementation failed, as her oxygen saturation on room air remained 90-91%. The patient continued to improve and engaged logically in conversation with the treatment team. She reported that her oxygen saturation normally trends between 89% and 91%. The hematology team evaluated the patient and ordered additional blood work and imaging studies. Echocardiography showed no pericardial effusion (Figure 1), and a CT scan of the chest revealed no pulmonary embolism (Figure 2). The blood work revealed G6PD deficiency and a variant hemoglobin present on electrophoresis, with elevated HbA₂ suggestive of an unstable beta-globin variant. The patient endorsed a paternal family history of G6PD deficiency. Direct Coombs test and autoimmune workup were unremarkable. Other results included: reticulocyte count: 6.63%, total bilirubin: 7.6 mg/dL, direct bilirubin: 0.68 mg/dL, lactate dehydrogenase (LDH): 249 U/L, and haptoglobin <10 mg/dL.

The patient continued to show clinical improvement, no longer reporting auditory hallucinations, and demonstrated good insight into her health condition. Her oxygen saturation on room air remained 91-92%. The medical, psychiatry, and hematology teams re-evaluated the patient and determined that she was stable for discharge. The patient was discharged home on Risperidone 2 mg PO bid, with follow-up appointments scheduled at the medicine, psychiatry, and hematology outpatient clinics.

## Discussion

Risperidone, when administered in overdose or in combination with other central nervous system-depressing agents, can lead to respiratory depression, manifesting as low oxygen saturation. An unanticipated delayed respiratory depression was reported following a risperidone overdose of more than 8 mg in the mid-2000s [11]. In the index case, the patient was not taking any other central nervous system-depressing agent. In a three-month study assessing the respiratory effects of risperidone, airway hyperreactivity was reported in two recruited patients, though there was no significant progression of respiratory disorder [12]. The authors suggested that, despite the small number of participants, risperidone may have secondary respiratory effects.

In this case, the patient had G6PD deficiency, a condition that can present with low oxygen saturation. G6PD deficiency is a potential cause of hemolysis [10]. One of the triggers of oxidative stress in G6PD-deficient individuals is the ingestion of fava beans, which can lead to hemolysis [13]. The ingestion of fava beans can also cause methemoglobinemia (MetHb), an abnormal variation of hemoglobin [13]. Both hemolysis and MetHb can cause low oxygen saturation [10]. Some of the hematological results for the index patient included: reticulocyte count 6.63%, total bilirubin 7.6 mg/dL, direct bilirubin 0.68 mg/dL, LDH 249 U/L, and haptoglobin <10 mg/dL. It is possible that the patient experienced hemolysis during this period. Risperidone has also been reported to cause anemia [14]. Risperidone-induced priapism was highlighted in 2022 during a review of case series [15]. Though the mechanism remains speculative, hemolysis is a possible contributing factor. Pancytopenia has also been documented during risperidone therapy [16].

Elevated HbA₂ levels, commonly seen in beta thalassemia, do not directly cause low oxygen saturation [16]. The index patient, who did not have beta thalassemia, had elevated HbA₂ suggestive of an unstable beta-globin variant. A hemoglobin variant is a recognized cause of low oxygen saturation, as reported in 2023 [17].

Although there appears to be no direct correlation between risperidone therapy and low oxygen saturation, the index patient had hematological features, namely G6PD deficiency and elevated HbA₂, that could contribute to low oxygen saturation. The multidisciplinary approach provided robust care, which was significant in the patient’s improvement during her clinical trajectory.

## Conclusions

In conclusion, this rare case underscores the importance of obtaining a detailed history following initial psychiatric stabilization and highlights the need for a holistic evaluation of any uncommon findings in patients receiving psychotropic medications. The patient showed clinical improvement during the course of treatment despite persistently low oxygen saturation, which, upon detailed history-taking, was found to be her baseline. Her elevated HbA₂, suggestive of an unstable beta-globin variant as a contributing factor to low oxygen saturation, provides valuable insight for future research.
